# Molecular self-assembly of nylon-12 nanorods cylindrically confined to nanoporous alumina

**DOI:** 10.1107/S2052252514020132

**Published:** 2014-10-21

**Authors:** Yan Cao, Hui Wu, Yuji Higaki, Hiroshi Jinnai, Atsushi Takahara

**Affiliations:** a Japan Science and Technology Agency, ERATO, Takahara Soft Interfaces Project, Fukuoka 819-0395, Japan; b Institute for Materials Chemistry and Engineering, Kyushu University, Fukuoka 819-0395, Japan

**Keywords:** molecular self-assembly, nanorods, selected-area electron diffraction, cylindrical confinement

## Abstract

Molecular self-assembly of nylon-12 rods in self-organized nanoporous alumina cylinders with two different diameters (65 and 300 nm) is studied with transmission electron microscopy (TEM) and wide-angle X-ray diffraction (WAXD) in symmetrical reflection mode. In a rod with a 300 nm diameter, the tendency of the hydrogen-bonding direction of a γ-form crystal parallel to the long axis of the rod is not clear because of weak two-dimensional confinement. In a rod with a diameter of 65 nm, the tendency of the hydrogen-bonding direction of a γ-form crystal parallel to the long axis of the rod is more distinct because of strong two-dimensional confinement. For the first time, selected-area electron diffraction (SAED) is applied in a transmission electron microscope to a polymer nanorod in order to determine the hydrogen-bond sheet and lamellar orientations. Results of TEM–SAED and WAXD showed that the crystals within the rod possess the γ-form of nylon-12 and that the *b* axis (stem axis) of the γ-form crystals is perpendicular to the long axis of the rod. These results revealed that only lamellae with 〈*h*0*l*〉 directions are able to grow inside the nanopores and the growth of lamellae with 〈*hkl*〉 (*k* ≠ 0) directions is stopped owing to impingements against the cylinder walls. The dominant crystal growth direction of the 65 nm rod in stronger two-dimensional confinement is in between the [−201] and [001] directions due to the development of a hydrogen-bonded sheet restricted along the long axis of the rod.

## Abstract 2

It has been revealed that in cylindrical nano-confinement, the hydrogen-bonding direction of nylon-12 crystals in the rod could self-assemble to be parallel to the long axis of the rod. The dominant growth direction and hydrogen-bonding direction of the γ-form crystal in the long axis of the rod has been revealed by TEM–SAED and WAXD.

## 1. Introduction   

Polymer crystallization under soft or hard cylindrical confinements has been widely studied during the past decade. The symmetry breaking caused by the two-dimensional confinement of the curved pore surface has introduced much interesting fundamental polymer physics in the phase separation of block copolymers (Nakagawa *et al.*, 2012 ▶; Dobriyal *et al.*, 2009 ▶; Zhu *et al.*, 2000 ▶; Sun *et al.*, 2006 ▶; Huang *et al.*, 2001 ▶; Sun *et al.*, 2004 ▶). As well as electro-spinning, nanolithography and phase separation of block copolymer, one-dimensional elongated rod-shaped nanomaterials made of organic molecules or polymers were also generated by using the self-organized nanoporous alumina (Wu *et al.*, 2012 ▶, Kim *et al.*, 2013 ▶, Chen *et al.*, 2012 ▶; Byun *et al.*, 2011 ▶; Choi *et al.*, 2013 ▶). The anodized alumina oxide (AAO) templates were prepared by a two-step anodization process (Masuda & Fukuda, 1995 ▶). In one-dimensional rod preparation, polymer melts or polymer solutions as a bulk reservoir were drawn into the nanoporous alumina by capillary force.

So far, limited species of thermoplastics have been fabricated into one-dimensional nanorods and only a small number of publications have tackled the crystallization of one-dimensional polymer nanorods (Steinhart, 2008 ▶). Polymer melt of polyvinylidene fluoride (PVDF) (Steinhart *et al.*, 2006 ▶), isotactic polypropylene (Duran *et al.*, 2011 ▶), polyethylene (PE) (Shin *et al.*, 2007 ▶), isotactic polystyrene (Wu *et al.*, 2013 ▶), syndiotactic polystyrene (sPS) (Wu *et al.*, 2007 ▶), poly(3-hexylthiophene) (Chen *et al.*, 2012 ▶), poly(vinylidene difluoride–trifluoroethylene) [P(VDF-TrFE)] (Shingne *et al.*, 2013 ▶), polyethylene oxide (Guan *et al.*, 2013 ▶; Suzuki *et al.*, 2013 ▶), *etc*. were fabricated into rod-shaped samples with diameters ranging from 10 nm to 300 nm within the AAO templates. In our work, nylon-12 was prepared into rods with diameters of 65 and 300 nm by using the methods mentioned above. In contrast to nylon-6, nylon-12 has wider applications in the automobile, coating and adhesive industries because it has a smaller hydrogen-bonded effect (Li & Goddard, 2002 ▶).

The crystal orientations of one-dimensional nanomaterials are normally characterized by wide-angle X-ray diffraction (WAXD), polarized Fourier transform infrared (FTIR)–ATR (attenuated total reflection) spectroscopy and TEM–SAED. WAXD and FTIR–ATR could be directly applied to polymer rods within the AAO template to investigate the crystal orientation of polymer rods in the AAO template (Steinhart, 2008 ▶). As for the crystallized PVDF rod-shaped samples under a hard cylindrical confinement, the 〈*hk*0〉 crystal orientations were observed along the long axis of the rod by WAXD experiments in reflection mode using a Philips X’pert MRD diffractometer and the 〈020〉 direction was the dominant crystal growth direction aligned with the pore axes (Steinhart *et al.*, 2006 ▶).

The effect of hydrogen bonding on the molecular self-assembly of polymers confined to nanoporous alumina has not yet been explored. One related area is the nanotube of a block copolymer [polyurethane (PU)-*b*-polytetramethylene oxide] prepared by AAO on the anisotropic crystal orientation of the PU nanowires (hard segment). Compared with the randomly distributed hydrogen-bond directions of PU nanowires inside the 270 nm diameter nanotube, the hydrogen-bond directions of PU nanowires inside the 60 nm diameter nanotube are parallel to the longitudinal direction of nanowires examined using FTIR–ATR (Park *et al.*, 2012 ▶).

For the first time, we use the TEM–SAED technique to investigate the molecular arrangement in polymer rods. In contrast to using WAXD and FTIR–ATR to obtain information based on a group of rods in the AAO template statistically, the significant advantage of the TEM–SAED technique is the ability to localize the electron beam to detect the morphology and structure of different individual rods that are released from the AAO template. The application of TEM–SAED in polymer rods may open the door to the analysis of the molecular arrangement of polymer nanorods.

The limitation of TEM–SAED applications in polymer nanorods was due to the rapid damage of rod-shaped samples under exposure to the strong electron beam. Only an electron diffraction (ED) experiment on sPS nanorods was carried out, showing that the crystalline structure is the orthorhombic β-form (Wu *et al.*, 2007 ▶).

In this study, we performed a series of TEM–SAED experiments on different individual nylon-12 nanorods in diffraction mode using the smallest condenser aperture, a suitable diffraction aperture and a beam current as low as possible. By comparing the reciprocal vectors of the SAED pattern with respect to the long axis of the rod in the real-space image, the crystal growth direction in the long axis of the rod could be determined.

WAXD in symmetrical reflection geometry was also directly applied to the AAO template to analyze the crystal growth direction in the long axis of the rod. Using symmetrical reflection geometry, only crystallographic planes parallel to the AAO template surface (perpendicular to the long axis of the rod) could be observed in the diffraction pattern. Both TEM–SAED and WAXD results show that the most common monoclinic (pseudohexagonal) γ-form crystals grow in the nylon-12 rod identified from SAED patterns in both the 300 nm and the 65 nm rod. The γ-form crystal orientation was discussed on two different length scales: lamellar (15–50 nm) and stem (0.5–2 nm) packing in two-dimensional confinement (Cheng, 2007 ▶; Cheng & Lotz, 2005 ▶).

Combining the SAED and WAXD results, we found that two-dimensional confinement of nylon-12 imposed by AAO significantly influences the hydrogen-bonding direction in the γ-form crystals inside the rod. In the 300 nm-diameter rod, the tendency of the hydrogen-bonding direction of the γ-form crystal parallel to the long axis of the rod is not clear because of weak two-dimensional confinement. In the 65 nm-diameter rod, the tendency of hydrogen-bonding direction of the γ-form crystal parallel to the long axis of the rod is more distinct because of strong two-dimensional confinement. To understand the molecular arrangement of polymers at the nanoscale could give guidance on how to manipulate or control the molecular orientation so as to fabricate the advanced polymer nanorod with anisotropic performance.

## 2. Experiment   

### 2.1. Materials   

Nylon-12 from UBE Industries Ltd was used as received in this study. The major microstructure characteristics of nylon-12 (*M*_w_ = 23000 and polydispersity, *M*_w_/*M*_*n*_, = 1.45) were determined by size exclusion chromatography at 313 K with a Waters 1515 HPLC system equipped with a refractive index detector using three polymethacrylate (PMMA)-based TSK gel columns (α-6000, α-5000 and α-4000; Tosoh Bioscience, Tokyo, Japan). The carrier solvent was 1,1,1,3,3,3-hexafluoro-2-propanol with a flow rate of 0.5 ml min^−1^. The calibration curve was established with PMMA standards.

### 2.2. Preparation of nylon-12 rods in AAO templates   

Self-ordered AAO templates with pore diameters of 300 nm and 65 nm were made by a two-step anodization process (Masuda & Fukuda, 1995 ▶). A schematic diagram of the fabrication of the nylon-12 rods and the sample preparation for TEM experiments is displayed in Fig. S1 in the supporting information.

### 2.3. Transmission electron microscopy   

Bright field (BF) morphologies and ED experiments were carried out with a Jeol-1200EX TEM at an acceleration voltage of 100 kV. In order to limit the beam damage to the sample, the smallest condenser aperture and a low-intensity electron beam were used and the rods were checked with a defocused beam in diffraction mode. TEM–SAED experiments were carried out by inserting a diffraction aperture. SAED experiments of nylon-12 300 and 65 nm rods were carried out for several tens of rods randomly chosen in the transmission electron microscope. The *d*-spacings were calibrated using thallium chloride (TlCl) as standard. The detailed sample preparation is illustrated in Fig. S1.

### 2.4. Scanning electron microscopy   

An Hitachi S-4300SE scanning electron microscope at an acceleration voltage of 5 kV was used to capture the images of AAO templates. All samples were coated with osmium before measurements in the microscope.

### 2.5. Wide-angle X-ray diffraction   

A Rigaku RINT 2500V diffractometer (Rigaku Denki Co., Ltd) with a Cu *K*α X-ray source at a voltage of 40 kV and a current of 200 mA in symmetrical reflection geometry was directly used to characterize the nylon-12 rods within the AAO template. The AAO template was placed on top of a glass substrate and the template surface was perpendicular to the plane of the incident and the reflected X-ray beams. In this symmetrical reflection geometry, only the crystallographic planes parallel to the AAO template surface (perpendicular to the long axis of the rod) could contribute to the reflections.

## 3. Results and discussion   

### 3.1. Morphologies of the nylon-12 300 and 65 nm rod   

**Figure 1:**
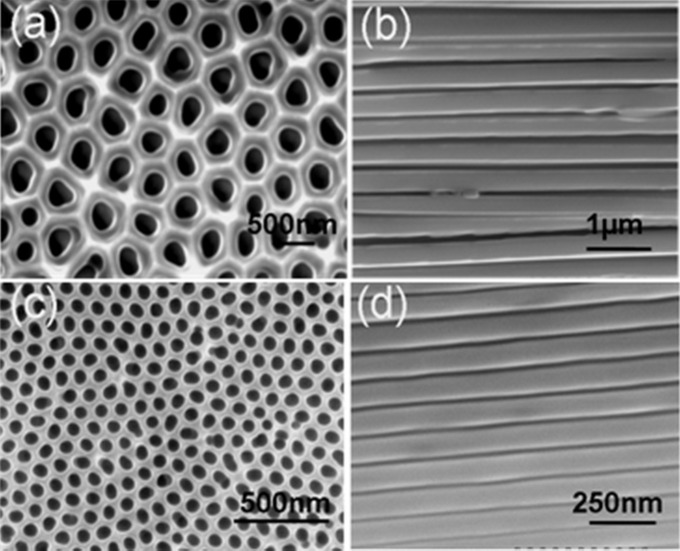
SEM images of the AAO templates. (*a*) Top and (*b*) side views of the AAO template with pore diameter of 300 nm, (*c*) top and (*d*) side views of the AAO template with pore diameter of 65 nm.

**Figure 2:**
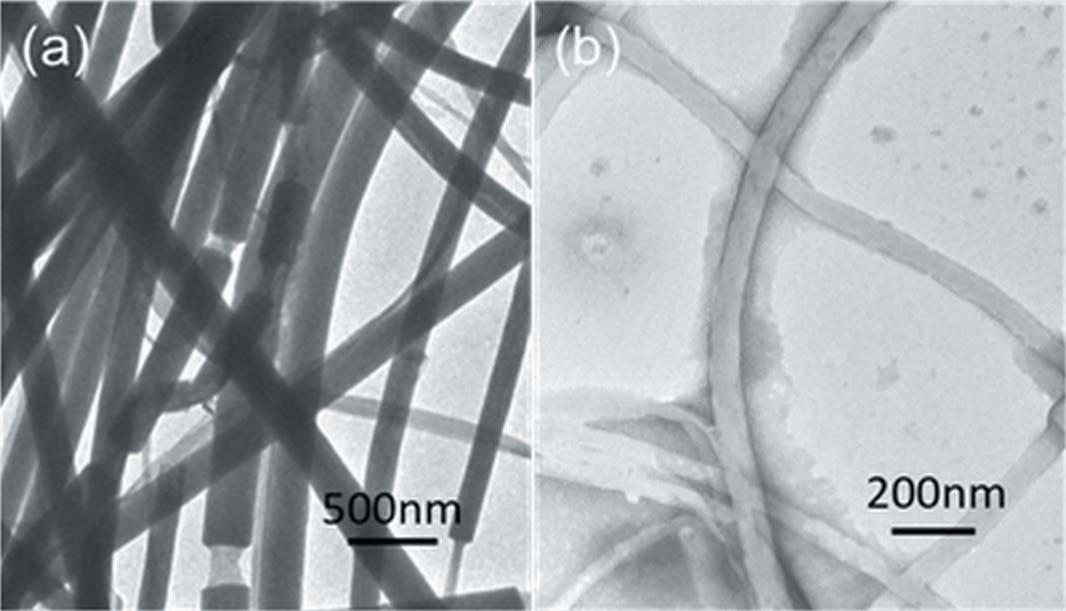
BF TEM images of the nylon-12 (*a*) 300 nm rod and (*b*) 65 nm rod.

Fig. 1 ▶ shows SEM micrographs of the top and side views of the AAO templates with pore diameters of 300 nm and 65 nm. The SEM images show that the nanopores of the AAO template are self-organized in an hexagonal packing. The BF TEM images in Figs. 2 ▶(*a*) and 2 ▶(*b*) show that the nylon-12 rods are released from the AAO templates, which, as expected, have diameters of 300 nm and 65 nm, respectively. The nucleation of nylon-12 under two-dimensional confinement is different from bulk heterogeneous nucleation. Differential scanning calorimetry results show that the homogeneous nucleation of nylon-12 occurs under two-dimensional confinement. The crystallization kinetics and thermodynamics of nylon-12 under two-dimensional confinement will be reported in another paper (Cao *et al.*, 2014 ▶).

### 3.2. Lamellar and stem packing of γ-form in the nylon-12 rods   

**Figure 3:**
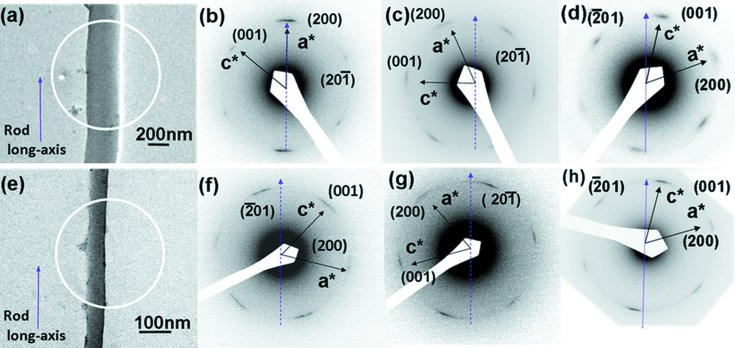
BF TEM micrograph (*a*) of a 300 nm rod and SAED patterns (*b*, *c*, *d*) with the [010] zone of γ-form crystals within the 300 nm rods. The crystal growth direction in the long axis of the rod is shown along (*b*) the [200] direction and in between (*c*) the [200] and [20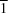] and (*d*) the [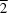
01] and [001] directions. BF TEM micrograph (*e*) of a 65 nm rod and SAED patterns (*f*, *g*, *h*) with the [010] zone of γ-form crystals within the 65 nm rod. The crystal growth direction is shown in between (*f*) the [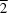
01] and [001], (*g*) the [200] and [20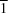] and (*h*) the [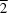
01] and [001] directions. Purple dashed lines denote the long axis of the rod.

Fig. S2 in the supporting information displays the one-dimensional WAXD patterns of nylon-12 rods measured in symmetrical reflection geometry. One strong reflection at 2θ = 21.4° with a *d*-spacing of 0.416 nm appears in WAXD patterns of nylon-12 rods (Fig. S2). SAED experiments were carried out for several tens of rods randomly chosen under TEM. Figs. 3 ▶(*b*)–3 ▶(*d*) and 3 ▶(*f*)–3 ▶(*h*) display SAED results of nylon-12 300 and 65 nm rods, respectively. We obtained diffractions with two *d*-spacings of 0.416 and 0.400 nm from SAED patterns of a nylon-12 rod (Fig. 3 ▶). Monobe & Fujiwara (1967 ▶) also viewed these two *d*-spacings in the ED pattern of a γ-form single crystal of nylon-12.

Both TEM–SAED and WAXD patterns show the characteristic reflection with a *d*-spacing of 0.416 nm for γ-crystals from nylon-12. No characteristic reflections of α-crystals from nylon-12 at *d*-spacings of 0.37 and 0.44 nm were observed in WAXD and TEM–SAED results. In general, the most common monoclinic (pseudohexagonal) γ-form is more stable than the monoclinic *α*-form (Dencheva *et al.*, 2005 ▶; Li *et al.*, 2003 ▶; Ramesh, 1999 ▶; Inoue & Hoshino, 1973 ▶; Rhee & White, 2002 ▶). Some reports claimed the γ-form of nylon-12 possessed an hexagonal structure because of one typical *d*-spacing of 0.416 nm. Our SAED results show that the γ-form of nylon-12 is monoclinic (pseudo-hexagonal) as proposed by Inoue & Hoshino (1973 ▶). The pseudo-hexagonal γ-form slightly deviates from the hexagonal structure due to close *d*-spacings of 0.416 and 0.410 nm (Inoue & Hoshino, 1973 ▶).

Using the cell features of the monoclinic (pseudo-hexagonal) γ-form (*a* = 0.938 nm, *b* = 3.22 nm (fiber axis), *c* = 0.487 nm, β = 121.5°), *d*_200_ is calculated to be 0.400 nm, and 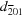, *d*_001_ is calculated to be 0.416 nm. Although the *d*-spacings *d*_(200)_ (0.400 nm) and *d*_(001)_ (0.416 nm) are close, they are easy to separate in the ED patterns. Therefore, this reflection with a *d*-spacing of 0.416 nm could be assigned to be either 001 or 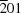 reflections of γ-form. The reflection with a *d*-spacing of 0.400 nm could be assigned to be the 200 reflection. The a* axis is deduced from the 200 diffraction spot and the c* axis is deduced from the 001 diffraction spot. The *c* axis could be deduced from the axis orientation of a* (a* 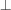 *c*). Based on a γ-form cell feature, the hydrogen-bonding direction parallel to the *c* axis could be deduced from the axis orientation of **c**.

Using the Scherrer (Scherrer, 1918 ▶) method, the crystallite size along the [001] or [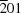] directions (parallel to the long axis of the rod) is estimated to be 7.6 nm for a 65 nm rod (supporting information, Fig. S2). The coherence length of 7.6 nm for the 65 nm rod is smaller than the pore diameter of 65 nm. Small crystallites of nylon-12 could grow in the nanopore with a diameter of 65 nm. A (20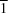) dark field TEM image of a 65 nm rod shows that nylon-12 crystallites spread in the whole rod (Cao *et al.*, 2014 ▶).

Owing to the close interplanar *d*-spacings (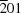), (001) and (200), the (200) reflection might include in this broad reflection. According to the symmetrical reflection geometry, the (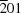), (001) and possibly (200) planes are parallel to the AAO template surface (perpendicular to the long axis of the rod). Therefore, based on WAXD results, the [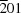] and [001] directions are crystal growth directions in the long axis of the rod. The [200] direction is the possible crystal growth direction in the long axis of the rod because of close *d*-spacings of (001), (200) and (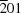).

To understand how the γ-form crystals grow within each rod, we carried out SAED experiments for several tens of nylon-12 rods randomly chosen under a tranmission electron microscope. The crystal growth direction in the long axis of the rod can be analyzed by looking at the particular reciprocal vectors of the SAED pattern with respect to the real-space rod image. Fig. 3 ▶(*a*) is a BF TEM image of a single nylon-12 300 nm rod, on which the SAED experiments were conducted. ED experiments on different 300 nm rods show three probable patterns as displayed in Figs. 3(*b*)–3(*d*). In Figs. 3(*b*)–3(*d*), the four weak inner arcs with a *d*-spacing of 0.416 nm are assigned as 001, 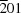, 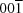 and 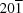 reflections of the γ-form. The other two outer strong arcs with *d*-spacing of 0.400 nm are assigned as 200 and 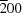 reflections. Fig. 3 ▶(*b*) shows that the crystal growth direction in the long axis of the rod is along the [200] direction. In Fig. 3 ▶(*c*), the crystal growth direction within the rod is in between the [200] and [20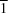] directions. On the other hand, in Fig. 3 ▶(*d*), the crystal growth direction in the long axis of the rod is in between the [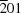] and [001] directions. Fig. 3 ▶(*e*) shows the BF TEM image of a single nylon-12 65 nm rod. ED experiments on different 65 nm rods show two probable patterns for the γ-form in nylon-12 as displayed in Figs. 3 ▶(*f*)–3 ▶(*h*). In Fig. 3 ▶(*f*), the crystal growth direction in the long axis of the rod is in between the [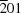] and [001] directions, and crystal growth direction in the long axis of the rod in Fig. 3 ▶(*g*) is in between the [200] and [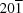] directions. In Fig. 3 ▶(*h*), the crystal growth direction in the long axis of the rod is also in between the [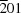] and [001] directions.

Therefore, the crystal growth directions in the long axis of the rod could be the [200], [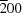], [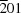], [001], [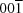] or [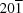] directions, which are summarized as the 〈*h*0*l*〉 directions in crystallography. Based on ED and WAXD results in the 300 nm rod, the probability of the [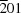] and [001] directions is slightly higher than the other two sets of crystal growth directions. In the 65 nm rod, the [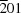] and [001] directions are the prevailing crystal growth directions compared with another two sets of crystal growth directions because of strong two-dimensional confinement. Therefore, the dominant crystal growth direction in the long axis of the rod of nylon-12 is in between the [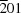] and [001] directions. The fast growth directions in the γ-form single-crystal is also in between the [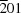] and [001] directions identified by Monobe & Fujiwara (1967 ▶). The dominant crystal growth direction was also reported in both PVDF and PE rod-shaped samples to be the [020] growth direction aligned with the long axis of the rod (Steinhart *et al.*, 2006 ▶; Maiz *et al.*, 2013 ▶).

**Figure 4:**
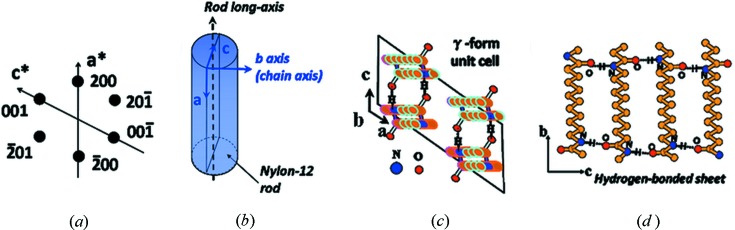
(*a*) Calculated ED pattern of the γ-form crystal from the [010] zone, (*b*) schematic diagram of chain axis of the nylon-12 γ-form crystal within the rod, (*c*) *ac*-plane projection of γ-form unit cell and (*d*) *bc*-plane projection of hydrogen-bonded γ-form sheets.

The calculated ED pattern of the γ-form crystal with the [010] zone is displayed in Fig. 4 ▶(*a*). The only existence of the (*h*0*l*) diffractions indicates that the *b * axis (stem axis) of these nylon-12 lamellae is *perpendicular* to the long axis of the rod as shown in Fig. 4 ▶(*b*). Fig. 4 ▶(*c*) is the top view of *ac*-plane projection of the unit cell and Fig. 4 ▶(*d*) is the side view of the *bc*-plane projection of the hydrogen-bonded sheet (Li *et al.*, 2003 ▶). Figs. 4(*c*) and 4(*d*) also show that the hydrogen-bonding direction of the γ-form crystal tends to be parallel to the *c* axis of the unit cell.

**Figure 5:**
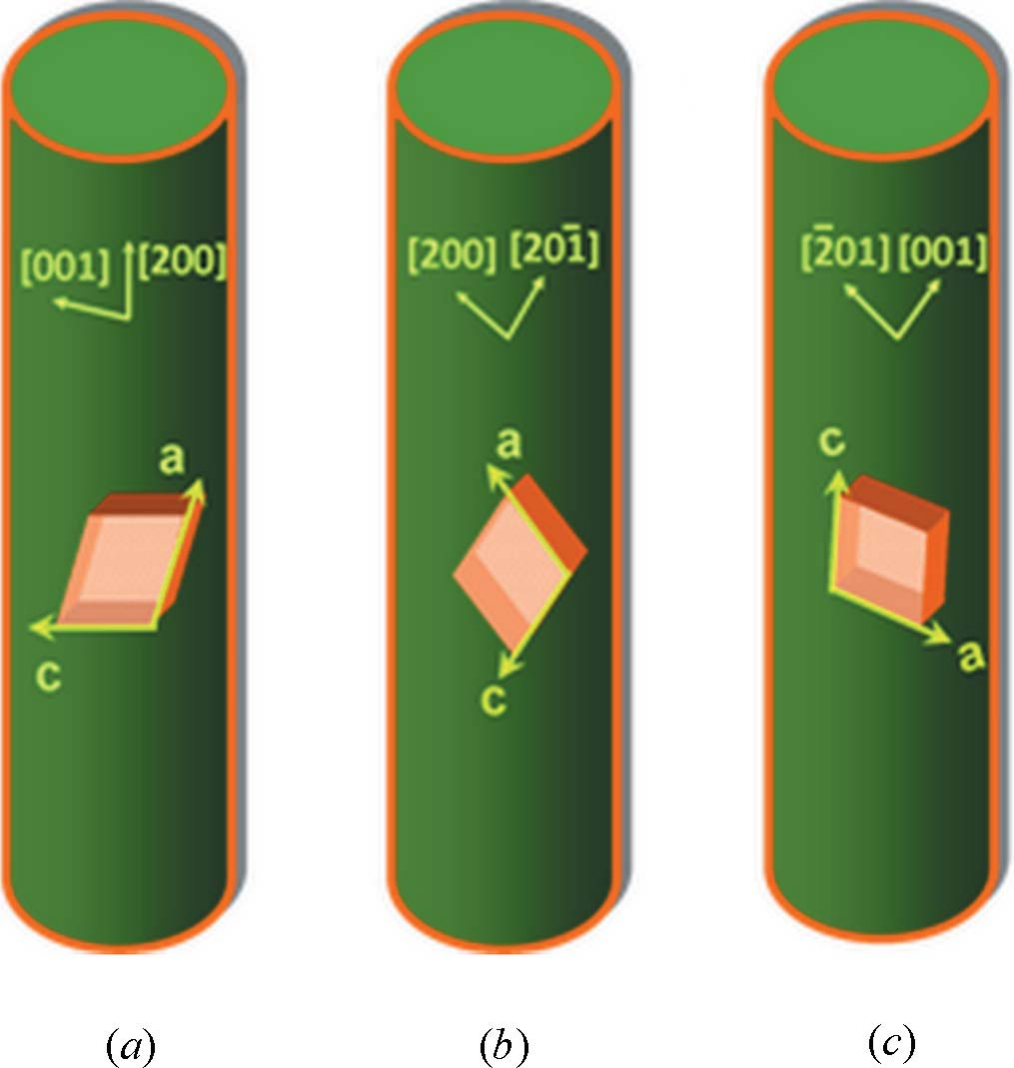
Three γ-form crystal growth directions in nylon-12 rods correspond to different γ-cell orientation with respect to the long axis of the rod. (*a*) The γ-form crystal growth direction in the long axis of the rod is along the [200] direction as determined by the SAED pattern in Fig. 3 ▶(*b*), or (*b*) in between the [200] and [20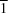] directions in Figs. 3 ▶(*c*) and 3 ▶(*g*), or (*c*) in between the [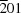] and [001] directions in Figs. 3 ▶(*d*), 3 ▶(*f*) and 3 ▶(*h*).

Figs. 5(*a*)–5(*c*) schematically display the three crystal growth directions in the long axis of the rod concluded from all of the ED experiments. The axis orientations in the upper part of cylinder show the crystal growth directions (reciprocal vectors in square brackets) in the long axis of the rod. The crystal growth directions are concluded from all of the SAED patterns of Fig. 3 ▶. According to the mathematical definition of a reciprocal lattice, the axis orientation of *a* and *c* could be deduced from the axis orientation of a* and c* (a* 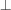 *c*, c* 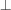 *a*), respectively. Therefore, the axis orientation of the unit cell, *a* and *c* axes in the real space, is displayed on the bottom part of cylinder. Thus, when the crystal growth direction in the long axis of the rod is along the [200] direction, the *c* axis of the γ-form unit cell is perpendicular to the long axis of the rod as displayed in Fig. 5 ▶(*a*). In Fig. 5 ▶(*b*), when the crystal growth direction in the long axis of the rod is in between the [200] and [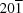] directions, the *c* axis of the γ-form unit cell was tilted with respect to the long axis of the rod. In Fig. 5 ▶(*c*), when the crystal growth direction in the long axis of the rod is in between the [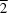
01] and [001] directions, the *c* axis of the γ-form unit cell tends to be parallel to the long axis of the rod.

**Figure 6:**
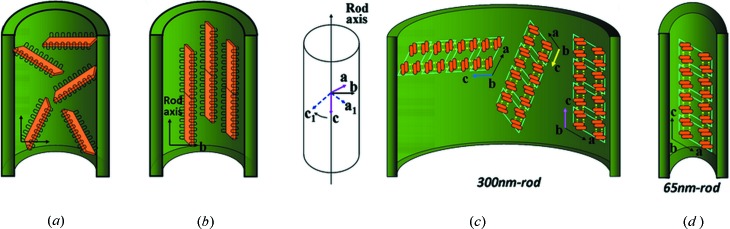
Schematic diagrams of the γ-form lamellae growth. (*a*) 〈*hkl*〉 directions (*k* ≠ 0) and (*b*) 〈*h*0*l*〉 directions within the rod. In the 300 nm rod (*c*), three hydrogen-bonding directions rotating within the *ac*-plane of the γ-form. (*d*) Packing of parallel chains of the γ-form within the 65 nm rod at the *ac*-plane projection.

As discussed above, two questions that arise are: why do the crystals inside the pore align with the 〈*h*0*l*〉 directions in the long axis of the rod and why was either the [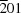] or [001] direction the dominant crystal growth direction aligned with the long axis of the 65 nm rod? The answers must be associated with the lamellar orientation and stem packing of the γ-form within the nylon-12 rod. If the stems of γ-form lamellae are tilted with respect to the long axis of the rod, lamellae grow inside the pore with the 〈*hkl*〉 directions (*k* ≠ 0) as shown in Fig. 6 ▶(*a*). Under this condition, the lamellar growth was easily stopped by the pore wall due to cylindrical confinement. Therefore, the stems (the *b* axis) of the γ-form lamellae are perpendicular to the long axis of the rod and then elongated lamellae with the 〈*h*0*l*〉 directions could develop in the long axis of the rod as shown in Fig. 6 ▶(*b*). Compared with the stem orientation in nylon-12 rod (stem axes are vertical to the cylinder axis), the stem axis in nylon-12 fibers with diameters of about a few microns are parallel to the cylinder axis (Inoue & Hoshino, 1973 ▶; Liu *et al.*, 2007 ▶).

As for the second question above, at a molecular level, the dominant crystal growth directions in the long axis of the rod may reflect the two-dimensional confinement to the hydrogen-bonding direction of nylon-12 rod. In the 300 nm rod, three hydrogen-bonding directions (the *c* axis) of the *ac* plane projection of the γ-form unit cell [Fig. 6 ▶(*c*) (right)] could be deduced from the three crystal growth directions in Figs. 5(*a*), 5(*b*) and 5(*c*), respectively. Fig. 6 ▶(*c*) (left view) shows that the hydrogen-bonding directions (*c* axis) of the γ-form unit cell in different 300 nm rods rotate within the *ac* plane without affecting the *b*-axis orientation which remains vertical to the long axis of the rod.

Based on the monoclinic γ-form cell feature proposed by Inoue & Hoshino (1973 ▶), the *b* axis (stem axis) is vertical to the *a* and the *c* axes. Since the hydrogen-bonding direction is parallel to the *c* axis based on the γ-form cell feature, the *b* axis (stem axis) is vertical to the hydrogen-bonding direction (*c* axis). The hydrogen-bonding direction (*c* axis) rotates within the *ac*-plane of the γ-form unit cell and still remains vertical to the stem axis (*b* axis) within the rod in Fig. 6 ▶(*c*). Therefore, the changes of hydrogen-bonding direction do not affect the *b*-axis orientation. As mentioned above, the three hydrogen-bonding directions in different 300 nm rods show less restriction to the γ-form crystal growth in the large rod. On the other hand, in the small 65 nm rod, based on the dominant crystal growth direction (in between the [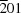] and [001] directions), the dominant hydrogen-bonding direction (the *c* axis) of the *ac*-plane projection of the γ-form unit cell, as shown in Fig. 6 ▶(*d*), tends to be nearly parallel to the long axis of the rod. Therefore, the hydrogen-bonded sheets of nylon-12 in the 65 nm rod were restricted from developing in the long axis of the rod because of stronger two-dimensional confinement.

## 4. Conclusions   

TEM–SAED and WAXD experiments in symmetrical reflection geometry were carried out to analyze the crystal growth direction in the long axis of nylon-12 rods imposed by two-dimensional confinement. Based on TEM–SAED and WAXD patterns of rod-shaped samples, the crystal orientation of nylon-12 rods was discussed at two different length scales: lamellar and stem packing.

SAED patterns taken from both the 300 nm and 65 nm nylon-12 rods displayed the 001, 200, 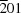 and 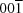, 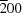, 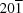 negative reflections, respectively, from the [010] zone of the γ-form crystal. The stems of γ-form lamellae in the nylon-12 rods are perpendicular to the long axis of the rod. In two-dimensional confinement, lamellae orientating with the 〈*h*0*l*〉 directions were grown in the pore. Lamellae growth in the 〈*hkl*〉 directions (*k* ≠ 0) was stopped by the pore wall.

As compared with the three hydrogen-bonding directions in the 300 nm rods, the hydrogen-bonding directions in 65 nm rods incline to be aligned with the long axis of the rod identified by the dominant crystal growth directions (the [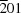] and [001] directions). This molecular arrangement in nylon-12 rods could provide guidance for manipulating the anisotropic performance of one-dimensional elongated rod-shaped nanomaterials.

## Supplementary Material

Fabrication and one-dimensional WAXD patterns of nylon-12 rods. DOI: 10.1107/S2052252514020132/zx5001sup1.doc

Fabrication and one-dimensional WAXD patterns of nylon-12 rods. DOI: 10.1107/S2052252514020132/zx5001sup2.pdf
