# Supplementary material for: Molecular self-assembly of nylon-12 nanorods cylindrically confined to nanoporous alumina
Source: IUCrJ. 2014 Oct 21;1(Pt 6):439–45. doi: 10.1107/S2052252514020132 (PMC4224462; doi:10.1107/S2052252514020132)
Supplement: Supplementary file 1 [file m-01-00439-sup1.doc]

[**IUCrJ**](http://journals.iucr.org/m)

**Volume 1 (2014)**

**Supporting information for article:**

[**Molecular self-assembly of nylon-12 nanorods cylindrically confined to nanoporous alumina**](http://dx.doi.org/10.1107/S2052252514020132)

**Yan Cao, Hui Wu, Yuji Higaki, Hiroshi Jinnai and Atsushi Takahara**

1. Methods for fabrication nylon-12 rod

Six steps of fabricating nylon-12 and sample preparation are described as following: Step 1 Putting nylon-12 film with a thickness about 200µm underneath the AAO template; Step 2 Heating samples above the melting temperature, *T_m_* (Tm=179.6^o^C)and annealed for 10 hours to prepare infiltrated-AAO sample; Step 3 Scratching off the film from the filled-AAO template surface and the AAO surface was polished by soft polishing paper ; Step 4 The scratched filled-AAO samples was heated above *T_m_* again and held for 10-15min (using FP-90 Mettler hot-stage), and then quenched to 160^o^C as fast as possible and kept for 12 hours. The filled-AAO sample was subsequently immersed into a 5wt% KOH solution about 4 hours; Step 5 Nylon-12 rods were released from the templates and rinsed by the deionized water; Step 6 Rods were picked up onto the copper grids for TEM experiments.

**
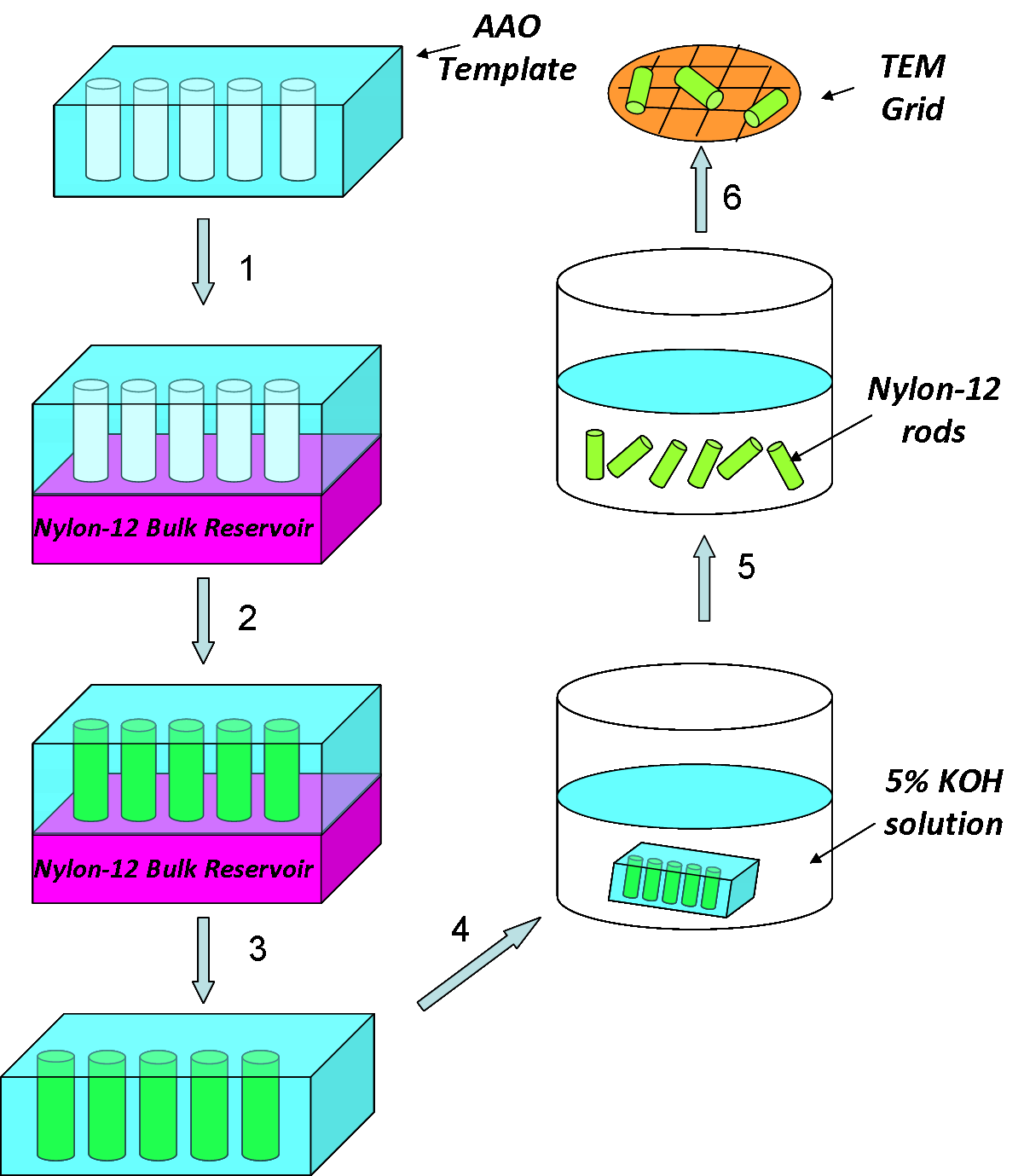
**

1. Fabricating of 1D nylon-12 rods and sample preparation for TEM measurement.
2. Wide-angle X-ray Diffraction (WAXD)

1. WAXD (symmetric reflection method) patterns of the nylon-12 rod within AAO with diameters of 300nm and 65nm.
